# Supplementary material for: Bioactive Compounds and Potential Health Benefits through Cosmetic Applications of Cherry Stem Extract
Source: Int J Mol Sci. 2024 Mar 27;25(7):3723. doi: 10.3390/ijms25073723 (PMC11011441; doi:10.3390/ijms25073723)
Supplement: Supplementary file 1 [file ijms-25-03723-s001.zip › ijms-2826881-supplementary.pdf]

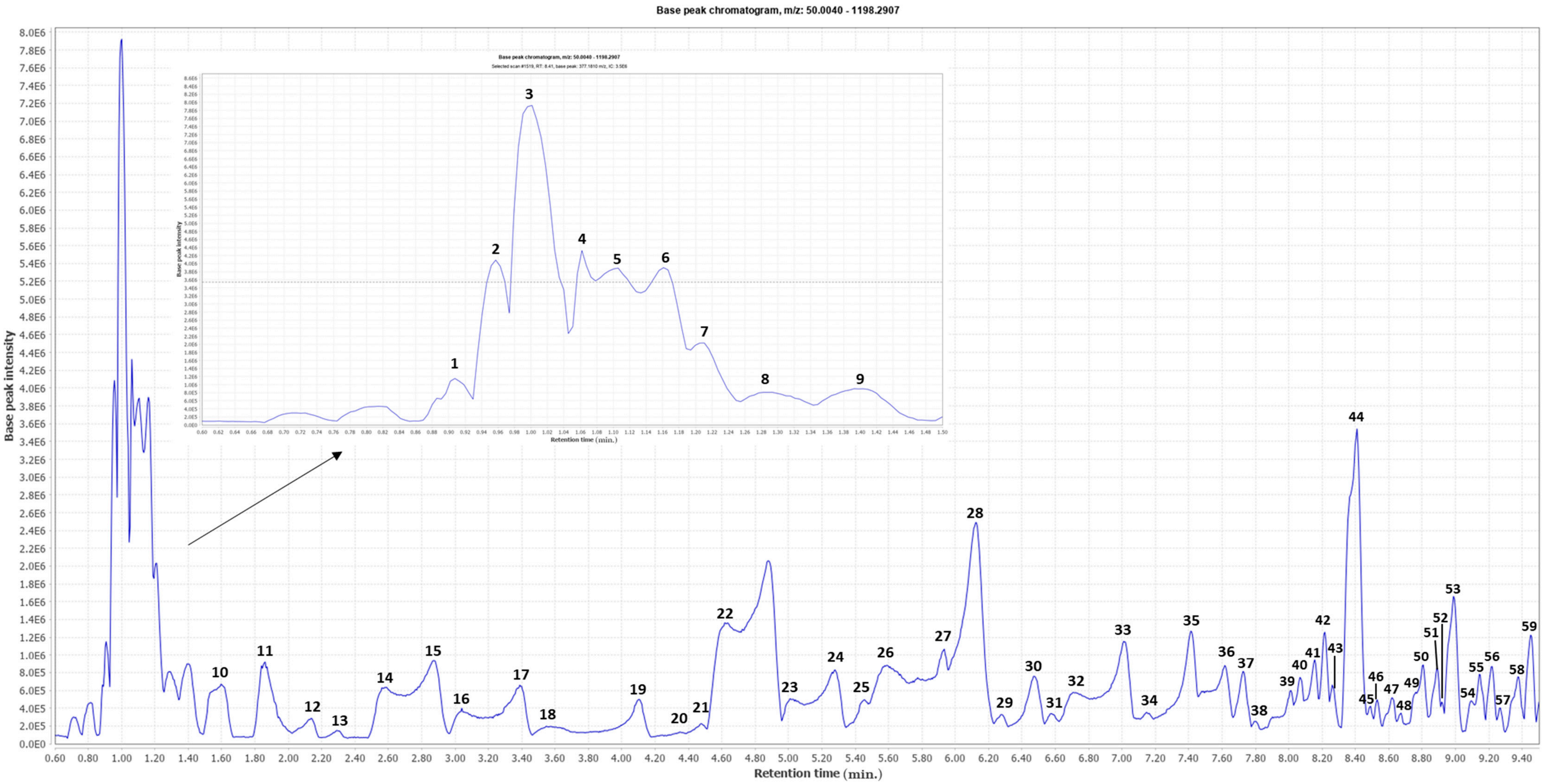
Base peak chromatogram, m/z: 50.0040 - 1198.2907
Base peak intensity
Retention time (min.)
Base peak chromatogram, m/z: 50.0040 - 1198.2907
Base peak intensity
Retention time (min.)
1 2 3 4 5 6 7 8 9
10 11 12 13 14 15 16 17 18 19 20 21 22 23 24 25 26 27 28 29 30 31 32 33 34 35 36 37 38 39 40 41 42 43 44 45 46 47 48 49 50 51 52 53 54 55 56 57 58 59

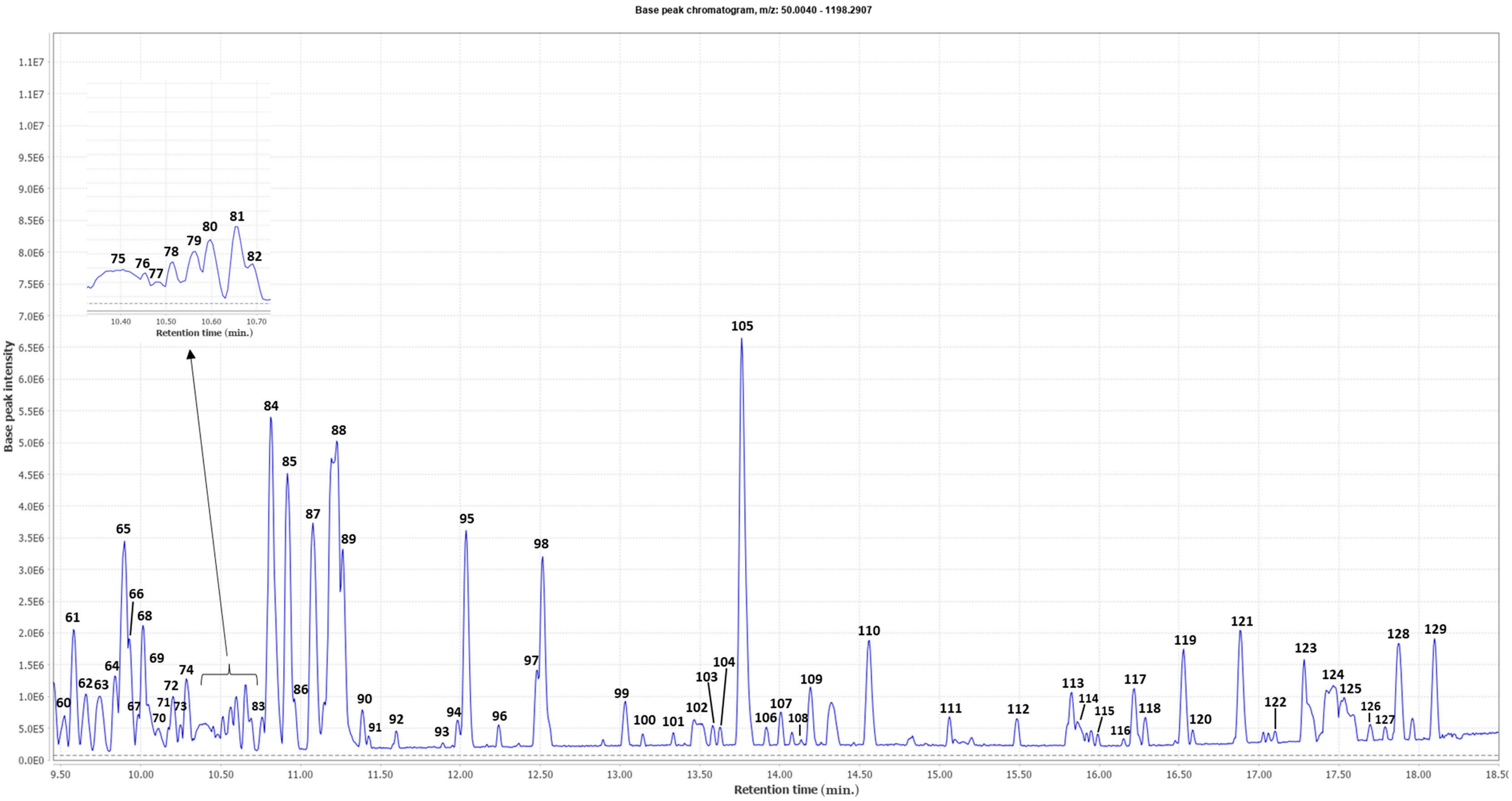

Base peak chromatogram, m/z: 50.0040 - 1198.2907
Base peak intensity
Retention time (min.)
0.0E0
5.0E5
1.0E6
1.5E6
2.0E6
2.5E6
3.0E6
3.5E6
4.0E6
4.5E6
5.0E6
5.5E6
6.0E6
6.5E6
7.0E6
7.5E6
8.0E6
8.5E6
9.0E6
9.5E6
1.0E7
1.1E7
1.1E7
9.50
10.00
10.50
11.00
11.50
12.00
12.50
13.00
13.50
14.00
14.50
15.00
15.50
16.00
16.50
17.00
17.50
18.00
18.50
10.40
10.50
10.60
10.70
75 76 77 78 79 80 81 82
60 61 62 63 64 65 66 67 68 69 70 71 72 73 74 83 84 85 86 87 88 89 90 91 92 93 94 95 96 97 98 99 100 101 102 103 104 105 106 107 108 109 110 111 112 113 114 115 116 117 118 119 120 121 122 123 124 125 126 127 128 129

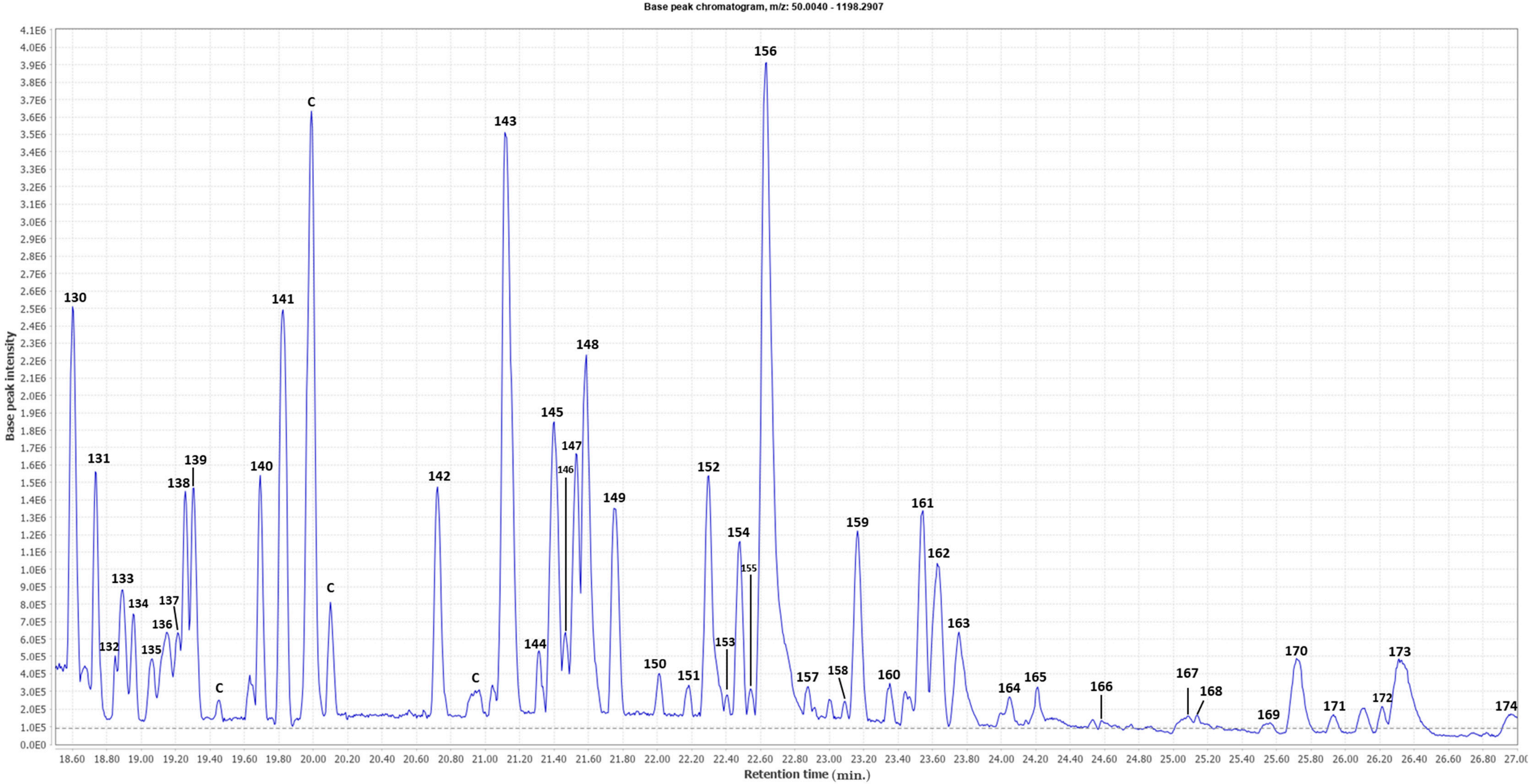

Figure S1: Base peak chromatogram from cherry stem extract; C: contaminant (signal detected also in blank samples).
